# Is there a relation between pre-sarcopenia, sarcopenia, cachexia and osteoporosis in patients with ankylosing spondylitis?

**DOI:** 10.1186/s12891-016-1155-z

**Published:** 2016-07-11

**Authors:** Abdellah El Maghraoui, François Bertin Ebo’o, Siham Sadni, Abderrahim Majjad, Toufik Hamza, Aziza Mounach

**Affiliations:** Rheumatology Department, Military Hospital Mohammed V, Rabat, University Mohammed V Souissi, PO Box: 1018, Rabat, Morocco

**Keywords:** Ankylosing spondylitis, Sarcopenia, Cachexia, Osteoporosis, DXA, Body composition

## Abstract

**Background:**

Osteoporosis is a well-known complication of ankylosing spondylitis (AS). However, data about body composition modifications and muscle performance showed conflicting results. The aim of the study was to determine the prevalence and risk factors of pre-sarcopenia, sarcopenia and cachexia in patients with AS and analyze its relationship with bone loss and symptomatic and severity parameters of the disease.

**Methods:**

Sixty-seven consecutive male patients with AS (mean age of 40.9 ± 11.0 years) and 67 healthy controls were studied. Body composition and bone mineral density (BMD) scans were obtained using DXA. The fat-free mass index (FFMI; fat-free mass divided by height squared) and the percent of fat mass (%FM) were calculated. Pre-sarcopenia was defined by low skeletal muscle mass (SMI <7.25 kg/m^2^), sarcopenia by the combined presence of the two following criteria: SMI <7.25 kg/m^2^ and a low muscle strength (handgrip strength <30 kg) or a low muscle performance (timed get-up-and-go test >10 s) and cachexia by a BMI <20 kg/m^2^ plus 3 from the 5 following parameters: anorexia, fatigue, handgrip strength <30 kg, CRP >5 mg/l, SMI <7.25 kg/m^2^.

**Results:**

Pre-sarcopenia, sarcopenia, cachexia, and osteoporosis prevalences were (50.4, 34.3, 11.9, and 16.0) respectively. Patients had a mean 3 kg significant decrease in FFM and a 1 kg/m^2^ decrease in appendicular mass vs. healthy controls. Pre-sarcopenia, sarcopenia and cachexia were significantly associated to higher BASDAI levels and low BMD.

**Conclusion:**

Our study showed that men with AS had a statistically significant reduction in total and appendicular lean mass that is related to higher disease activity and significantly associated to bone loss.

**Electronic supplementary material:**

The online version of this article (doi:10.1186/s12891-016-1155-z) contains supplementary material, which is available to authorized users.

## Background

Sarcopenia is a term that was used first to define age related skeletal muscle wasting. Now, it is used to describe all kinds of loss of muscle tissue and function whatever the cause is (aging, chronic diseases, or low protein-energy intake and physical inactivity) [1]. In the context of chronic inflammatory disorders, some authors called cachexia the accelerated loss of skeletal muscle to differentiate it from the age related sarcopenia. However, the spectrum of body composition changes in disease states varies widely from a minimal weight loss related to skeletal muscle wasting to an extreme state of loss of fat and muscle in refractory cachexia (as in cancer), including the particular case of the normal or high BMI of sarcopenic obesity, that combines high muscle loss with increased fat mass (as reported in rheumatoid arthritis) [2]. Sarcopenia, as defined by muscle loss and dysfunction, is a common feature of all chronic inflammatory diseases and involves impairment of either contractile, metabolic and endocrine functions of skeletal muscle [3] and is related to elevated circulating proinflammatory cytokines and especially tumor necrosis factor (TNF). As also patients with ankylosing spondylitis (AS) show elevated levels of these cytokines [4], sarcopenia would be expected in this population.

Sarcopenia diagnosis relies currently on determination of muscle mass, strength and physical performance. Assessment of muscle mass can be done using several techniques such as anthropometry, bioimpedance analysis (BIA), dual energy X-ray absorptiometry (DXA), computed tomography (CT) scan and magnetic resonance imaging (MRI). Although CT and MRI are considered as gold standards for estimating muscle mass in research, DXA is the preferred alternative method for research and clinical use for body composition assessment [5]. Muscle strength can be measured reliably using the handheld dynamometer in upper extremities while physical performance can be assessed using several tests such as the gait speed, the Timed Get-Up-and-Go and the Short Physical Performance Battery. The latter includes standing balance, gait speed, and chair rises (sit-to-stand). It is noteworthy however to remind that no specific definition of sarcopenia in inflammatory chronic disease populations exists.

Many studies of body composition in patients with AS are published and did not consistently shown a reduction in muscle mass [6–8]. These conflicting findings may be related to the differences in the studied populations (such as disease duration and severity) and the variety of methods used to assess body composition. In the other hand, it is well established that osteoporosis, even in the early stages of AS, is a common feature that seems to be related to disease severity [9–11].

Therefore, the aim of this study was to determine the prevalence and risk factors of pre—sarcopenia/sarcopenia/cachexia in a group of patients with AS and analyze its relationship with osteoporosis and symptomatic and severity parameters of the disease.

## Methods

### Patients and healthy controls

The study group consisted in 67 male patients with AS who fulfilled the modified New York criteria for the classification of AS and who presented consecutively to our department between September 2014 and July 2015. A group of 67 age-matched (within 2 yrs) healthy subjects was recruited from the same local population (hospital staff members and their family members, visitors…etc.) to serve as controls according to exclusion criteria. A subjects’ written consent was obtained according to the Declaration of Helsinki and the study was approved by our local ethics committee (Military Hospital Mohammed V, Rabat). Exclusion criteria were the presence of a history of neuroendocrine disorders (thyroid, parathyroid disorders, anticonvulsant usage etc.), chronic renal or liver diseases, systemic high dose steroid use, and excessive alcohol intake. The following data were collected for all the subjects: age, height, weight, and body mass index (BMI). The time elapsed between the onset of first AS related symptoms and enrollment defined disease duration. Disease activity and the functional consequences of the disease were assessed by the Bath AS disease activity index (BASDAI) and the Bath AS functional index (BASFI), respectively. Erythrocyte sedimentation rate (ESR) and C-reactive protein (CRP) were assessed using standard laboratory techniques. Sacroiliitis was assessed on anteroposterior pelvic radiographs and graded according to the New York Scale. Spine structural damage was assessed by the Stoke AS Spinal Score (SASSS) in which each corner is scored for the presence of squaring, sclerosis, erosions, syndesmophytes and bridging syndesmophytes with a maximal score of 72.

### Bone mineral density measurements

The DXA scans were obtained by standard procedures using Lunar Prodigy Vision machine. All BMD measurements were carried out by 2 experienced technicians. Daily quality control was carried out by measurement of a Lunar phantom which showed at the time of the study stable results. The coefficient of variation of the phantom precision was 0.08 and the reproducibility assessment in clinical practice showed in a previous study a smallest detectable difference of 0.04 g/cm^2^ (spine) and 0.02 (hips) [12]. Patient BMD was measured at the lumbar spine (anteroposterior projection at L1–L4) and at the femurs (i.e., femoral neck, trochanter, and total hips) and the classification system of the World Health Organization (WHO) was applied, defining osteoporosis as T-score ≤ −2.5 and osteopenia as −2.5 < T-score ≤ −1 according to the lowest T-score of the L1–4 lumbar spine, femur neck, or total femur.

### Body composition parameters assessment

All anthropometric measures were taken following standard procedures by the same investigator (FBE). The subjects were weighed to the nearest 0.1 kg, and standing height was measured to the nearest 0.1 cm and BMI was then calculated from weight/height^2^ (kg/m^2^). In accordance with WHO standards, individuals with BMI values <18.5 kg/m^2^ were considered underweight, between 18.5 and 24.9 as normal, 25 and 29.9 as overweight and values greater than 30 indicated obesity [13]. Mid upper arm circumference and waist circumference were measured using a plastic, inelastic, flexible belt-type measuring tape to the nearest 0.5 cm.

Body composition (total and regional fat mass and lean mass) was measured with total body DXA using the same machine. The whole body scan used the DXA system’s automated software, which provided compositional estimates of legs, arms, trunk, head, and whole body. Scans were performed with the subject wearing light indoor clothing. The precision of soft tissue analysis for a Lunar Prodigy is 1 % for fat-free mass (FFM) and 2 % for fat mass (FM) [14]. FFM and FM were expressed in absolute kg, and FM also as percentage of total mass. The normal reference value for FM% is 20 % to 30 % for women and 12 % to 20 % for men [15]. Fat free mass index (FFMI, kg/m^2^), fat mass index (FMI, kg/m^2^) and skeletal mass index (SMI, kg/m^2^, where appendicular skeletal muscle mass is standardized using the square of the individuals’ height) were also calculated.

### Muscle strength and performance assessment

Maximal voluntary grip strength of the dominant hand was measured with a Grip-A dynamometer (Takey, Kiki Kogyo, Japan). This assessment could be easily done as only 4 patients among the study population had peripheral involvement and none of the patients had hand joints involvement.

Global muscle performance was assessed by the Timed Get-Up-And-Go test: the subject rises from a chair, walks 3 meters, turns around, returns to the chair, and sits down. The subject was instructed to: “Sit with your back against the chair and your arms on the arm rests. On the word ‘go’, stand upright, then walk at your normal pace to the line on the floor, turn around, return to the chair, and sit down.“ The stopwatch was started on the word ‘go’ and stopped when the subject returned to the starting position.

### Mini Nutritional Assessment

The MNA (0–30 points) is a dietary questionnaire assessing the number of meals, food and fluid intake and autonomy of feeding. It is a subjective assessment of self-perception of health and nutrition which also includes questions related to lifestyle, medication and morbidity. MNA classifies individuals with adequate nutritional status (>23.5 points), with risk for malnutrition (17–23.5 points) and with malnutrition (<17 points) [16].

### Definition of pre-sarcopenia, sarcopenia and cachexia

As a consequence of the lack of a simple and commonly accepted definition for these conditions, we choose arbitrarily one from the published definitions:

- Pre-sarcopenia was defined according to Baumgartner definition [17] by SMI <7.25 kg/m^2^.
- Sarcopenia was defined by the combined presence of the two following criteria according to the European Working Group on Sarcopenia in Older People (EWGSOP) [5]: a low muscle mass (SMI <7.25 kg/m^2^) and a low muscle strength (assessed by a handgrip strength <30 kg) or a low muscle performance (assessed by a timed get-up-and-go test >10 s).
- Cachexia was defined according to the International Working Group on Sarcopenia (IWGS) definition [18] by a BMI <20 kg/m [2] plus 3 from the 5 following parameters: anorexia, fatigue, handgrip strength <30 kg, CRP >5 mg/l, FFMI <7.25 kg/m^2^.

### Statistical analysis

The study was conducted on several steps. Step one consisted on the description of the study population. We compared in step 2 anthropometric and densitometric (BMD and body composition) variables between patients and healthy controls. In step 3, 4 and 5, we compared patients with or without pre-sarcopenia, sarcopenia and cachexia. A regression binary analysis was conducted in step 6 where the dependant variable was the presence of pre-sarcopenia and the independent variables the potential risk factors. And finally, to study the potential impact of TNF inhibitors (TNFi), in step 7, we compared patients taking or not TNFi.

Statistics Package for Social Sciences (SPSS Inc., Chicago, IL) was used for statistical analyses. Results are expressed in mean ± SD for quantitative variables and n (%) for qualitative variables.

## Results

### Subject characteristics

Subject characteristics are shown in Table 1. The mean ± SD (range) for age, disease duration and BASDAI score were 40.9 ± 11.0 years (18–66), 9.3 ± 7.9 years (1–36) and 3.7 (range 0–8.6) respectively. The proportion of patients with “active disease” according to the BASDAI (score of ≥4) was 32 (47.8 %). Twenty two (32.8 %) patients used TNFi since a mean duration of 2.3 years (range 0.25 – 7).

**Table 1:** Demographic characteristics and clinical features (symptomatic and structural disease severity parameters) in our study population with AS

			Minimum	Maximum
Age (yrs): m (SD)	40.7	(11.0)	18	66
Disease duration (yrs): m (SD)	9.3	(7.9)	1	36
BASDAI: m (SD)	3.7	(2.4)	0	8.6
BASMI: m (SD)	2.8	(5.5)	0	36
BASFI: m (SD)	40.5	(28.1)	0	83
BAS-G: m (SD)	17.5	(22.7)	0	72
ESR (mm/H): m (SD)	27.1	(21.0)	2	78
CRP (mg/l) : m (SD)	15.3	(25.1)	0.3	168.0
SASSS: m (SD)	10.7	(15.7)	0	72
Anti-TNF use : n (%)	22	(32.9)		
Anti-TNF use duration (yrs): m (SD)	2.3	(1.7)	0.25	7

*CRP* C-reactive protein, *ESR* erythrocyte sedimentation rate, *SASSS* Stoke Ankylosing Spondylitis Spinal Score

### Patients vs. healthy controls

Table 2 shows all of the body composition data for the patients and controls. Patients had a mean 3 kg significant decrease in FFMI and a 1 kg/m^2^ decrease in SMI. There was also a significant difference in lumbar spine and total hip bone mass and T-scores. Whereas there was 16 % among the patients that has osteoporosis (T-score below −2.5 in either the lumbar spine or the total hip) and 50.7 % that has pre-sarcopenia according to the definition of Baumgartner (low skeletal muscle mass: SMI <7.25 kg/m^2^), only 3 % of the controls had osteoporosis and 28 % had pre-sarcopenia. None of our patients had history of clinical fractures. Moreover, Vertebral fractures were assessed using VFA in 33 patients among the study population and showed mild fractures in only 3 patients (data not shown).

**Table 2:** Comparison of clinical variables, bone and body composition densitometric data in patients with AS and a healthy control group

	AS patients *N* = 67		Controls *N* = 67		*P*
Age (yrs): m (SD)	40.7	(11.0)	40.9	(11.0)	NS
Height (m): m (SD)	1.72	(0.08)	1.74	(0.08)	NS
Weight (kg): m (SD)	72.4	(13.2)	77.0	(12.0)	NS
BMI (kg/m^2^): m (SD)	25.3	(4.0)	24.1	(3.4)	NS
Fat-free mass (kg): m (SD)	49.2	(6.3)	52.0	(6.3)	0.014
Fat-free mass index (kg/m^2^): m (SD)	16.3	(2.0)	17.2	(1.9)	0.007
Fat mass (kg): m (SD)	21.2	(10.5)	22.8	(9.7)	NS
Fat mass index (kg/m^2^): m (SD)	6.9	(3.1)	7.5	(3.7)	NS
Appendicular mass (kg): m (SD)	22.2	(3.0)	23.4	(3.3)	0.033
Appendicular mass index (kg/m^2^): m (SD)	7.4	(0.8)	7.7	(0.9)	NS
Lumbar spine BMD (g/cm^2^): m (SD)	1.19	(0.1)	1.12	(0.1)	0.023
Lumbar spine T-score: m (SD)	−0.64	(2.5)	−0.10	(2.5)	0.019
Total hip BMD (g/cm^2^): m (SD)	0.97	(0.1)	1.08	(0.1)	<0.0001
Total hip T-score: m (SD)	−0.56	(1.3)	0.42	(1.1)	<0.0001
Osteoporosis any site: n (%)	13	(19.4 %)	2	(3.0)	0.002
Pre-sarcopenia: n (%)	33	(49.3)	19	(28.4)	<0.01

*BMI* body mass index, *SASSS* Stoke Ankylosing Spondylitis Spinal Score

### Determinants of pre-sarcopenia, sarcopenia and cachexia

Tables 3, 4, and 5 show the comparison between patients with and without pre-sarcopenia, with and without sarcopenia and with and without cachexia respectively. Globally, they showed that higher BASDAI levels, lower lumbar spine and hip BMD and T-scores and higher prevalence of osteoporosis are significantly associated to pre-sarcopenia, sarcopenia and cachexia.

**Table 3:** Comparison of anthropometric, clinical and densitometric variables between patients with AS with and without pre-sarcopenia (Baumgartner definition)

	AS patients with pre-sarcopenia *N* = 33	AS patients without pre-sarcopenia *N* = 34	*P*
Age (yrs): m (SD)	42.1 (11.6)	40.7 (10.7)	NS
Height (m): m (SD)	1.71 (0.06)	1.73 (0.06)	NS
Weight (kg): m (SD)	64.6 (11.0)	76.9 (11.9)	<0.0001
BMI (kg/m^2^): m (SD)	21.6 (3.6)	25.7 (3.7)	<0.0001
Disease duration (yrs): m (SD)	9.4 (9.1)	9.1 (6.6)	NS
Lumbar spine BMD (g/cm^2^): m (SD)	1.077 (0.23)	1.164 (0.14)	NS
Lumbar spine T-score: m (SD)	−1.05 (3.4)	−0.22 (1.2)	0.032
Total hip BMD (g/cm^2^): m (SD)	0.925 (0.13)	1.026 (0.23)	0.017
Total hip T-score: m (SD)	−0.89 (1.4)	−0.22 (1.1)	0.017
Osteoporosis: n (%)	10 (30.3)	3 (8.8)	0.0033
BASDAI (0–10): m (SD)	4.4 (2.4)	3.3 (2.5)	0.003
BASMI (0–10): m (SD)	2.8 (2.2)	2.8 (2.7)	NS
BASFI (0–100): m (SD)	45.5 (26.5)	35.0 (25.5)	NS
BAS-G (0–100): m (SD)	15.7 (16.8)	19.1 (22.6)	NS
Hand grip strength (kg): m (SD)	22.5 (7.0)	24.3 (9.8)	NS
Timed get-up-and-go test (sec) < 10 sec: n (%)	23 (74.2)	24 (75.0)	NS
Arm circumference (cm): m (SD)	26.8 (1.9)	30.5 (3.9)	<0.0001
Waist circumference (cm): m (SD)	85.7 (10.8)	97.1 (11.4)	<0.0001
MNA test (0–17): m (SD)	0.29 (0.4)	0.20 (0.3)	NS
SASSS (0–72): m (SD)	11.8 (16.7)	10.0 (15.6)	NS
CRP (mg/l): m (SD)	20.1 (10.1)	10.6 (10.1)	NS
ESR (mm/H): m (SD)	33.0 (22.3)	21.2 (18.4)	NS
Patients taking TNFi: n (%)	13 (28.9)	9 (42.9)	NS

*BMI* body mass index, *CRP* C-reactive protein, *ESR* erythrocyte sedimentation rate, *MNA* Mini Nutritional Assessment, *SASSS* Stoke Ankylosing Spondylitis Spinal Score

**Table 4:** Comparison of anthropometric, clinical and densitometric variables between patients with AS with and without sarcopenia according to EWGSOP definition

	AS patients with sarcopenia *N* = 23	AS patients without sarcopenia *N* = 44	*P*
Age (yrs): m (SD)	42.3 (11.6)	40.1 (10.7)	NS
Height (m): m (SD)	1.71 (0.06)	1.73 (0.06)	NS
Weight (kg): m (SD)	64.6 (11.0)	77.9 (11.9)	<0.0001
BMI (kg/m^2^): m (SD)	21.6 (3.6)	25.7 (3.7)	<0.0001
Disease duration (yrs): m (SD)	11.6 (9.1)	8.6 (6.6)	NS
Lumbar spine BMD (g/cm^2^): m (SD)	1.092 (0.23)	1.134 (0.14)	NS
Lumbar spine T-score: m (SD)	−0.97 (3.4)	−0.47 (1.2)	0.032
Total hip BMD (g/cm^2^): m (SD)	0.914 (0.13)	1.006 (0.23)	0.017
Total hipT-score: m (SD)	−0.96 (1.4)	−0.35 (1.1)	0.017
Osteoporosis: n (%)	7 (30.4)	4 (9.1)	0.038
BASDAI (0–10): m (SD)	4.4 (2.4)	3.4 (2.5)	0.002
BASMI (0–10): m (SD)	2.9 (2.2)	2.7 (2.7)	NS
BASFI (0–100): m (SD)	44.5 (26.5)	38.0 (25.5)	NS
BAS-G (0–100): m (SD)	19.7 (16.8)	16.1 (22.6)	NS
Hand grip strength (kg): m (SD)	19.5 (7.0)	25.3 (9.8)	NS
Timed get-up-and-go test (sec) < 10 sec: n (%)	14 (66.7)	33 (75.0)	NS
Arm circumference (cm): m (SD)	26.8 (1.9)	30.5 (3.9)	0.002
Waist circumference (cm): m (SD)	85.7 (10.8)	94.1 (11.4)	<0.0001
MNA test (0–17): m (SD)	0.34 (0.4)	0.19 (0.3)	NS
SASSS (0–72): m (SD)	14.3 (18.7)	9.5 (14.6)	NS
CRP (mg/l): m (SD)	21.8 (10.1)	11.6 (10.1)	NS
ESR (mm/H): m (SD)	31.0 (22.3)	24.2 (18.4)	NS
Patients taking TNFi: n (%)	11 (33.3)	11 (32.4)	NS

*BMI* body mass index, *CRP* C-reactive protein, *ESR* erythrocyte sedimentation rate, *MNA* Mini Nutritional Assessment, *SASSS* Stoke Ankylosing Spondylitis Spinal Score

**Table 5:** Comparison of anthropometric, clinical and densitometric variables between patients with AS with and without cachexia according to the IGWS definition

	AS patients with cachexia *N* = 8	AS patients without cachexia *N* = 59	*P*
Age (yrs): m (SD)	36.5 (13.6)	41.5 (10.7)	NS
Height (m): m (SD)	1.73 (0.06)	1.72 (0.06)	NS
Weight (kg): m (SD)	53.7 (11.0)	75.2 (11.9)	<0.0001
BMI (kg/m^2^): m (SD)	21.6 (3.6)	26.7 (3.7)	<0.0001
Disease duration (yrs): m (SD)	8.3 (9.1)	9.4 (6.6)	NS
LS BMD (g/cm^2^): m (SD)	1.150 (0.23)	0.892 (0.14)	<0.0001
LS T-score: m (SD)	−2.45 (3.4)	−0.38 (1.2)	<0.0001
TH BMD(g/cm^2^): m (SD)	0.856 (0.13)	0.991 (0.23)	0.037
TH T-score: m (SD)	−1.45 (1.4)	−0.44 (1.1)	0.016
Osteoporosis: n (%)	6 (75.0)	7 (11.9)	<0.0001
BASDAI (0–10): m (SD)	5.4 (2.4)	3.5 (2.5)	0.007
BASMI (0–10): m (SD)	3.5 (2.2)	2.7 (2.7)	NS
BASFI (0–100): m (SD)	52.3 (26.5)	35.0 (25.5)	NS
BAS-G (0–100): m (SD)	29.5 (16.8)	15.9 (22.6)	NS
Hand grip strength (kg): m (SD)	19.1 (7.0)	23.8 (9.8)	NS
Get-up and go test (sec) < 10 sec: n (%)	6 (75.0)	41 (74.5)	NS
Arm circumference (cm): m (SD)	25.8 (1.9)	29.5 (3.9)	<0.0001
Waist circumference (cm): m (SD)	78.4 (10.8)	92.8 (11.4)	<0.0001
MNA test (0–17): m (SD)	0.62 (0.4)	0.19 (0.3)	0.008
SASSS (0–72): m (SD)	8.3 (4.7)	10.6 (16.6)	NS
CRP (mg/l): m (SD)	39.2 (10.1)	11.6 (10.1)	0.013
ESR (mm/H): m (SD)	44.1 (22.3)	24.3 (18.4)	0.031
Patients taking TNFi: n (%)	21 (35.6)	1 (12.5)	NS

*BMI* body mass index, *BMD* bone mineral density, *CRP* C-reactive protein, *ESR* erythrocyte sedimentation rate, *LS* lumbar spine, *MNA* Mini Nutritional Assessment, *SASSS* Stoke Ankylosing Spondylitis Spinal Score, *TH* total hip

Table 6 shows regression binary analysis where pre-sarcopenia as defined by Baumgartner (SMI <7.25 kg/m^2^) was the dependent variable: BASDAI was the only variable significantly associated to pre-sarcopenia.

**Table 6:** Multiple regression analysis with pre-sarcopenia as defined by Baumgartner as the dependant variable

	Exp (B)	95 % CI	*p*
Age	1.008	0.941 – 1.080	NS
Disease duration	1.036	0.958 – 1.121	NS
BASDAI	1.050	1.002 – 1.086	0.029
CRP	1.017	0.967 – 1.070	NS

### Impact of TNFi treatment

Comparison between patients taking or not TNFi (Table 7) showed that patients taking TNFi had a statistically significant longer disease duration and higher FMI and lumbar spine BMD and T-scores. No difference was observed in muscle mass, muscle strength or performance tests or symptomatic or structural severity parameters of AS.

**Table 7:** Comparison of anthropometric, clinical and densitometric variables between patients with AS taking or not TNF inhibitors

	AS patients not taking TNFi *N* = 45	AS patients taking TNFi *N* = 22	*P*
Age (yrs): m (SD)	39.5 (11.4)	43.7 (11.5)	NS
Height (m): m (SD)	1.73 (0.06)	1.70 (0.06)	NS
Weight (kg): m (SD)	70.6 (14.2)	76.7 (13.3)	NS
BMI (kg/m^2^): m (SD)	23.3 (4.5)	25.7 (4.2)	NS
Disease duration (yrs): m (SD)	6.8 (6.0)	14.1 (8.9)	0.0001
FFMI: m (SD)	16.3 (1.8)	16.2 (2.0)	NS
FMI: m (SD)	5.9 (3.3)	9.0 (3.5)	0.001
Pre-sarcopenia as defined by Baumgartner : n (%)	23 (51.1)	11 (50.0)	NS
Sarcopenia as defined by EWGSOP: n (%)	12 (27.3)	9 (40.9)	NS
Cachexia as defined by IWSG : n (%)	7 (15.6)	1 (4.5)	NS
LS BMD (g/cm2): m (SD)	1.08 (0.19)	1.19 (0.19)	0.029
LS T-score: m (SD)	−0.90 (1.6)	−0.07 (1.3)	0.034
TH BMD(g/cm2): m (SD)	0.986 (0.18)	0.945 (0.13)	NS
TH T-score: m (SD)	−0.50(1.1)	−0.70 (1.0)	NS
Osteoporosis: n (%)	10 (22.2)	1 (4.5)	NS
BASDAI: m (SD)	3.7 (2.3)	3.6 (2.8)	NS
BASMI: m (SD)	2.7 (52.5)	3.0 (2.5)	NS
BASFI: m (SD)	39.5 (29.8)	41.0 (28.8)	NS
Hand grip strength (kg): m (SD)	23.1 (9.2)	23.1 (9.1)	NS
Get-up and go test (sec): m (SD)	3.4 (0.5)	2.0 (0.5)	NS
Arm circumference (cm): m (SD)	28.0 (3.3)	29.9 (3.8)	NS
Waist circumference (cm): m (SD)	88.3 (12.2)	97.5 (10.7)	0.004
MNA test: m (SD)	0.33 (0.4)	0.09 (0.2)	0.015
SASSS: m (SD)	11.6 (18.8)	7.0 (5.2)	NS
CRP (mg/l): m (SD)	17.0 (30.2)	11.3 (9.9)	NS
ESR (mm/h): m (SD)	29.3 (23.1)	22.3 (15.1)	NS

*BMI* body mass index, *BMD*: bone mineral density, *CRP*: C-reactive protein, *ESR* erythrocyte sedimentation rate, *LS* lumbar spine, *MNA* Mini Nutritional Assessment, *SASSS* Stoke Ankylosing Spondylitis Spinal Score, *TH* total hip

## Discussion

This study showed that men with AS had statistically significant reduction in total (3 kgs) and appendicular lean mass. These results are concordant with those of some previous DXA studies on the effects of AS on body composition: one study found a statistically non-significant reduction of 3 kg of total lean mass [6, 19] and the other study found a higher statistically significant difference of 6 kgs [20]. This lean mass loss seems to be related to higher disease activity and inflammation and significantly associated to bone loss.

As early as in the third century B.C., Hippocrates described the wasting syndrome associated to chronic diseases. Indeed, several metabolic abnormalities secondary to chronic diseases of multifactorial origin are observed. The term sarcopenia, which is derived from the Greek words sarx (flesh) and penia (poverty), describes a condition characterized by loss of muscle mass and muscle strength [1, 21]. Although sarcopenia is primarily a disease of the elderly, it may develop secondarily to conditions that can be seen in younger patients such as inflammatory diseases. As the muscle mass and muscle strength in younger individuals is high before it is affected by these disorders, muscle mass and muscle strength loss secondary to such disorders is usually thought to be functionally less relevant. Diagnosis of sarcopenia is based on the combined presence of a low muscle mass and a low muscle strength and/or performance. Cachexia, which is derived from the Greek words kako`s (bad) and he Ïxis (condition), has been defined as a syndrome of multifactorial origins characterized by severe body weight, fat and muscle loss and increased protein catabolism due to underlying disease(s). Cachexia is clinically relevant since it increases patients’ morbidity and mortality [22, 23]. Among the contributory factors to the onset of cachexia we can list anorexia and metabolic alterations, i.e. increased inflammatory status, increased muscle proteolysis, impaired carbohydrate, protein and lipid metabolism. Inflammation does play a crucial role in its pathogenesis and its presence allows for cachexia identification. Cachexia is linked to the disease activity of the chronic inflammatory diseases through the effects of proinflammatory cytokines such as tumor necrosis factor-α (TNF-α), interleukin 1 (IL-1), and IL-6. TNF-α (formerly called cachectin), a pivotal cytokine in rheumatic diseases, plays an important role in the development of cachexia. Skeletal muscle mass loss is considered the most clinically relevant phenotypic feature of cachexia, irrespective of the underlying causative disease [24].

In patients with RA, several studies, although not all, have shown evidence for a decreased FFM compared to healthy controls [25–27] often associated with increased FM and thus, with little or no weight loss, and a maintained body mass index. This combined condition has been called “rheumatoid cachexia”. In patients with AS, evidence is however limited: Toussirot et al. [6] did not found any difference in FFM or FM, as measured with DXA, between patients and controls. In the study of Sari et al. [7], using BIA, no difference in FFM between patients and controls was noted, but a lower percentage of FM was observed in male patients compared to healthy controls. The first study to show a lower appendicular and total lean mass (that was related to lower functional strength) was reported by Marcora et al. [20] in a series of male patients with AS. Another study conducted by Plasqui et al. [8] found no difference in FFM, and also not when corrected for height (FFMI) or expressed as a percentage of total body mass. In this study, the FFMI was even slightly higher in patients and interestingly; the observed values of the FFMI correspond to those of a large sample of healthy subjects.

Furthermore, most of previous studies have not reported the effects of AS on regional body composition. This is important since it has been shown that the appendicular lean mass (arms and legs) is a better proxy measure of total body skeletal muscle mass than total lean mass or FFM [28]. In addition, trunk lean mass could be related to spine immobility rather than the metabolic consequences of systemic inflammation. Moreover, it has been shown that body composition assessment using DXA including the trunk might be slightly affected by the kyphosis of the spine [29].

We found that AS-related sarcopenia was associated to low BMD in this cohort. We have already highlighted the link between lean mass loss and osteoporosis in patients with AS [30]. We showed that bone loss is common in AS and may be observed in early disease and that it is linked to persistent inflammation [31]. Many studies showed a positive impact of treatment with TNF inhibitors on BMD in patients with AS [32, 33]. However, whether TNF inhibitors have an effect on muscle loss is still controversial.

Anti-TNF-α therapy induces a significant and sustained reduction in clinical disease activity and systemic inflammation, and improves measures of disability in AS. In theory, anti-TNF therapy is expected to be effective to treat or prevent cachexia in patients with rheumatic disorders. As expected, infusions of anti-TNF-α antibodies in animal models have shown anticachectic effects [34]. Recently, Marcora et al. reported the first randomized controlled trial of anti-TNF therapy for cachexia in RA, but did not observe changes in weight and body composition induced by this treatment in this population [35]. Briot et al. have shown in a 2-year study of 106 patients with Spondyloarthropathies receiving anti-TNF therapy that a significant increase in weight of 2.2 kg occurred in 2 years (mostly due to a significant gain in FM) in parallel with an increase in BMD (+5.8 % at the lumbar spine and +2.26 % at the femur) [36]. This is concordant with the data of our study where one third of our patients were taking TNF inhibitors (mean: 2.3 years) and which show that patients taking TNF inhibitors has a significantly higher FM and FMI.

Our study has strengths and limitations. The assessment of body composition and BMD was carefully conducted using standard procedures of acquisition. The main limitation lies in the chosen definitions of pre-sarcopenia, sarcopenia and cachexia. However, as no consensual definitions exist, we used definitions developed by groups interested in sarcopenia/cachexia study and based on published cut-offs. Another limitation is the lack of data about vitamin D status which was not included in the study protocol. Further longitudinal studies are warranted to better evaluate the association between bone and muscle loss in AS and the long term effect of TNF inhibitors.

## Conclusions

Our study showed that men with AS had a statistically significant reduction in total and appendicular lean mass compared to healthy age-matched controls. This lean mass loss seems to be related to higher disease activity and significantly associated to bone loss. Patients with AS with important weight loss should be assessed using body composition analysis. TNF inhibitors may have a role in reducing muscle wasting.

## Abbreviations

AS, Ankylosing spondylitis; BASDAI, Bath AS disease activity index; BASFI, Bath AS functional index; BIA, bioimpedance analysis; BMD, bone mineral density; BMI, body mass index; CRP, C-reactive protein; CT, computed tomography; DXA, dual energy X-ray absorptiometry; ESR, Erythrocyte sedimentation rate; FFM, fat-free mass index; FM, fat mass; FMI, fat mass index; IWGS, International Working Group on Sarcopenia; MNA, Mini Nutritional Assessment; MRI, magnetic resonance imaging; SASS, Stoke ankylosing spondylitis Spinal Score; SMI, skeletal mass index; TNF, tumor necrosis factor; TNFi, tumor necrosis factor inhibitors; WHO, World Health Organization

## Additional file

Additional file 1: SPA cachexie Hommes &T anonym. (XLSX 544 kb)
